# Binding patterns of glycine receptor autoantibodies are related to clinical syndromes

**DOI:** 10.1186/s40478-025-02082-0

**Published:** 2025-07-31

**Authors:** Inken Piro, Anna-Lena Wiessler, Eleni Kakavela, Betül Baykan, Erdem Tüzün, Carmen Villmann, Claudia Sommer

**Affiliations:** 1 Department of Neurology, University Hospital Würzburg, Würzburg, Germany; 2 Institute for Clinical Neurobiology, University of Würzburg, Würzburg, Germany; 3 Department of Neurology and Clinical Neurophysiology, EMAR Medical Center, Istanbul, Türkiye; 4 Department of Neuroscience, Institute of Experimental Medicine, Istanbul University, Istanbul, Türkiye

**Keywords:** Glycine receptor, Autoantibodies, Stiff person syndrome, Binding pattern

## Abstract

Patients diagnosed with the rare autoimmune disease Stiff Person Syndrome (SPS) or the more severe form Progressive Encephalomyelitis with Rigidity and Myoclonus (PERM) as well as patients with encephalitis or epilepsy may harbor autoantibodies against synaptic molecules, for example the glycine receptor (GlyR). These autoantibodies interfere with inhibitory signal transmission, which causes a variety of symptoms. How the underlying autoantibody associated pathomechanisms contribute to the variability of clinical presentations, is so far not understood. In this study, binding patterns of GlyR autoantibodies from patients with SPS, PERM and epilepsy to murine central nervous system (CNS) tissue samples were analyzed for disease- and patient-specificity patterns. Twelve GlyR autoantibody positive patients were grouped by the patients’ primary diagnoses. Serum samples from these SPS, PERM and epilepsy patients were evaluated for autoantibody binding on transfected HEK-293 cells and murine spinal cord and various brain tissue samples. Autoantibody binding was further verified by co-localization with commercial antibodies binding to the GlyR and the synaptic marker synaptophysin. Immunochemistry revealed GlyRα1-specific autoantibody binding for all included patients on transfected HEK-293 cells and in the grey matter of murine spinal cord sections. Other CNS regions of enhanced autoantibody accumulation however varied among the groups of SPS, PERM and epilepsy patients and also within groups. Similarly, autoantibody deposits were detected in GlyR expressing higher brain areas for each patient. Even if variations between labeled areas and cell layers were rather patient-specific than group-specific, functionality of the labeled areas aligned with the patients’ impaired functions. Labeled areas and cell layers differing between patients could thereby explain the variability of symptomatology between and within the diseases. The observed diversity suggests a necessity for a personalized approach correlating patient-specific autoantibody properties, phenotype and treatment approaches.

## Introduction

The presence of glycine receptor (GlyR) autoantibodies (aAb) in patients’ blood and cerebrospinal fluid (CSF) has been associated with a variety of clinical syndromes, including Stiff Person Syndrome (SPS) and Progressive Encephalomyelitis with Rigidity and Myoclonus (PERM) [5], also summarized as stiff person spectrum disorder (SPSD) [8], but also with epilepsy and encephalitis [9, 25, 36]. Around 20% of SPS patients present with aAb against the GlyR, sometimes additionally to other aAb [25, 29]. About 50% of PERM patients harbor GlyR aAb. Main symptoms in SPS patients are spasms and stiffness of skeletal muscles [10, 34].

In the adult, GlyRs are located at pre- and postsynaptic sites mainly in spinal cord and brainstem. Additionally, GlyR subunits are also expressed in brain areas such as hippocampus, amygdala, thalamus and hypothalamus as well as in cerebellar cell layers [3, 21, 24, 27, 39]. Glycine signaling plays an important role in inhibitory signal transmission as its activation causes chloride ion influx into the cells, generating inhibitory postsynaptic potentials. aAb binding has been demonstrated to alter ion channel function by decreasing inhibitory signal transmission [9, 32, 41] which may explain spasms and stiffness of skeletal muscles observed in patients by altering the controlled firing at the nerve-muscle circuit. Rauschenberger et al. described a common autoantibody binding epitope ^29^A-^62^G (numbers refer to immature protein) in the far N-terminal region of the GlyRα1 subunit [32]. However, also other GlyRα subunits [5] or the GlyRβ subunit are targets of GlyR aAb in some patient samples [41].

Patient GlyR aAbs bind the postsynaptic pentameric GlyR which most likely consists of four α-subunits and one β-subunit according to findings on receptor stoichiometry [45, 47]. In a recent study, additional binding of GlyR aAbs to presynaptic homomeric GlyRs was described [42]. The subtype composition of homo- and heteropentameric receptor assemblies differs in dependence of the areas of the central nervous system (CNS) where the receptor is expressed [24, 31, 39].

The reported cases of GlyR aAb associated diseases show a high variability of clinical presentation and thus a diversity of combined symptoms. The underlying mechanisms that may explain the symptomatic variability in clinical phenotypes still needs to be elucidated.

A way to investigate different disease presentations is to study the binding patterns of the aAbs to tissue of the CNS. So far, tissue-based detection methods scarcely found application since GlyR aAb binding in tissue appeared unreliable due to lack of feasible protocols [11, 33]. The main difficulty is that GlyR aAb mostly bind to conformational epitopes of the receptor, which are not available in fixed tissue. Therefore, we developed a method using fresh frozen tissue samples and brief application of very mild fixation reagent that still allows aAb binding and visualization.

With this, we were able to identify individual GlyR aAb binding patterns in tissue-based assays. We compared binding patterns and clinical syndromes from patients with GlyR aAbs and the clinical picture of SPS, PERM, and epilepsy.

## Materials and methods

### Patients

Serum samples from 12 patients previously determined as seropositive for anti-GlyR aAbs were used in this study. Seven of the patients (patients 1 to 7) were diagnosed with SPS, two with PERM (patients 8 and 9) and three with childhood-onset epilepsy (patients 10 to 12; [17]). Clinical data are given in Table 1. SPS patients had increased muscle tone causing walking disabilities and falls. The latter were often associated with an exaggerated startle response. Some patients additionally suffered from anxiety. Patients 1, 5, 8, 11 and 12 were included in previous functional studies [32, 41, 42], patient 7 in a case report [13]. Patient 8 had a rapid evolution of symptoms, including double vision and somnolence that made him wheelchair bound within a few weeks. Patient 9 had an encephalopathy with prominent fatigue and seizures that had initially been diagnosed as psychogenic. All three patients with childhood-onset epilepsy had focal seizures evolving to bilateral tonic-clonic seizures.

**Table 1:** Clinical and demographic data of patients. Patients 1, 5, 7, 8, 11 and 12 were included in previous studies [13, 32, 41, 42]

Pat	Diagnosis	Age at diagnosis	Duration at blood withdrawal	Main symptoms	GlyRα1 ab titer	Other GlyR aAbs	Additional Antibodies
1	SPS	46	15	lower limb stiffness, spasticity and paresis, falls, startle, unsteady walking	1:1000	α2: pos. α3: neg.	GAD: pos.
2	SPS	13	40	painful muscle spasms with sudden falls and walking disability; minor rigidity; enhanced anxiety	1:25	α2: neg. α3: pos.	amphiphysin: neg. GAD: neg.
3	SPS	12	2	n.m.	1:50	α2: neg. α3: neg.	amphiphysin: neg. GAD: neg.
4	SPS	9	54	Painful spasms, walking disability and sudden falls, startle, neuromuscular hypertrophy, anxiety disorder	1:10	α2: pos. α3: neg.	amphiphysin, GAD, VGKC: all neg.
5	SPS	18	27	swallowing difficulties; stiffness, numbness, walking disability, intermittent hyposthesia; anxiety	1:1000	α2: neg. α3: neg.	amphiphysin: neg. GAD: neg.
6	SPS	69	3	Stiffness, spastic, painfull spasms, fasciculations; paraneoplastic manifestation	1:25	α2: neg. α3: neg.	n.m.
7	SPS	36	22	Chronic fatigue, lock jaw, painful muscle spasms; stiff lower limbs with walking disabilities and sudden falls;	1:50	α2: pos. α3: pos.	GAD: neg.
8	SPS/PERM	54	16	brainstem myoclonus, exaggerated startle, sensitive to minor auditory and tactile stimuli, abnormal eye movements with diplopia and nystagmus	1:1000	α2: pos. α3: neg.	amphiphysin: neg. GAD: neg.
9	Encephalitis/ PERM	18	0	Dissociative anxiety disorder, functional seizures, distractible walking disability	1:2000	α2: pos. α3: neg.	amphiphysin: neg., GAD: neg.
10	Focal epilepsy	3	16	focal seizures with impairment of conciousness, focal seizure evolving to bilateral convulsive seizure	1:500	α2: neg. α3: neg.	NMDAR, LGI1, CASPR2 GABABR, AMPAR, DPPX, GAD: all neg.
11	Focal epilepsy	16	17	focal seizures with impairment of conciousness, focal seizure evolving to bilateral convulsive seizure	1:500	α2: pos., α3: neg.	NMDAR, LGI1, CASPR2 GABABR, AMPAR, DPPX, GAD: all neg.
12	Mesial temporal lobe epilepsy	9	41	focal seizures with impairment of conciousness,	1:25	α2: neg. α3: neg.	NMDAR, LGI1, CASPR2 GABABR, AMPAR, DPPX, GAD: all neg.

VGKC = voltage gated potassium channel complex; # serum provided by H.-M. Meinck

GlyR aAb titers ranged from 1:10 to 1:2000 determined by immunofluorescence on live GlyRα1 transfected HEK-293 cells. For the purpose of identifying similarities and differences between pathomechanisms that cause similarly severe phenotypes in the patients, aAb concentrations were not equalized. Instead, the serum samples were all used in the same dilutions, irrespective of the initial antibody titer.

During diagnostic procedures, most of the patients were additionally tested for other neuronal aAb (Table 1). Of the evaluated patients, one SPS patient (patient 1) tested positive for GAD aAbs in addition to those directed against the GlyR.

### Cell line

For in vitro experiments, the human cell line HEK-293 (Human Embryonic Kidney cells; CRL-1573; ATCC – Global Bioresource Center, Virginia, U.S.) was used. Minimum essential medium (Life Technologies, Massachusetts, U.S.) was supplemented with 10% fetal bovine serum, L-glutamine (2 mM), 100 U/ml penicillin and 100 µg/ml streptomycin for cells to grow in at 37 °C and 5% CO_2_.

### Cell transfection

For evaluation of GlyR specificity of aAb positive sera, HEK-293 cells were transiently transfected by a calcium-phosphate precipitation method. Cells reached a confluency of 50–75% around 24 h after seeding of 200,000 cells on glass cover slips in a 35 mm cell culture dish. GlyRα1 was co-transfected with eGFP in a cDNA ratio of 1:1 with 1 µg of each plasmid DNA. DNAs were supplemented with 2.5 M CaCl_2_, 0.1x TE buffer and 2x HBS buffer (50 mM HEPES, 12 mM glucose, 10 mM KCl, 280 mM NaCl, 1.5 mM Na_2_HPO_4_). After 20 min of incubation at room temperature (RT, ∼21 °C), the mix was applied to the cells. The medium was exchanged after 4–6 h, and cells were used for experiments 48 h after transfection.

### Immunocytochemistry

*For live cell staining*, transfected cells were incubated with patient sera diluted 1:50 or a commercial monoclonal antibody against GlyRα1 (mouse: 146111 or rabbit: 146118, Synaptic Systems, Göttingen, Germany) diluted 1:500 in supplemented minimum essential medium for one hour. After fixation for 10 min using 4% paraformaldehyde (PFA) with 4% sucrose in phosphate buffered saline (PBS) pH 7.4, cells were washed three times by dipping them into PBS and then blocked with 5% normal goat serum (NGS) in PBS for 30 min. Another washing step was followed by incubation with secondary antibodies goat-anti-human-IgG-Cy3 (109-165-003, Dianova, Hamburg, Germany) goat-anti-mouse-Cy3 (115-165-003, Dianova, Hamburg, Germany) or goat-anti-rabbit-Cy3 (111-165-003, Dianova, Hamburg, Germany,) 1:500 in blocking solution for 1 h in the dark. Cells were washed again with PBS. Cell nuclei were stained with 4’,6-diamidino-2-phenylindole (DAPI) 1:5,000 in PBS for 5 min. After a last washing step with PBS, cells were dipped into H_2_O_dest_ and mounted on microscope slides with mowiol mounting medium. All incubations were performed at RT.

*For staining with commercial pan-GlyRα antibody* (mouse monoclonal 146011, Synaptic Systems) and to test human sera on fixed and permeabilized cells, HEK-293 cells were fixed with 4% PFA and 4% sucrose in PBS pH 7.4 for 20 min at 4 °C, washed three times with PBS, then blocked and permeabilized using 5% NGS and 0.3% Triton-X_100_ in PBS for 30 min at RT and finally incubated with primary antibodies diluted 1:500 in 5% NGS in PBS for 1 h at RT. For fluorescent detection of commercial pan-GlyRα or human aAb GlyRα1 binding, cells were washed again and incubated with secondary antibodies and DAPI as described above.

### Tissue preparation

After decapitation, brain and spinal cord tissue was dissected from wildtype C57BL/6 mice, freshly frozen in Tissue-Tek^®^ O.C.T.™ Compound (Sakura Finetec Germany GmbH, Staufen im Breisgau, Germany) and stored at -80 °C. Brain and spinal cord were sliced into 9-µm transversal (spinal cord) or coronal (brain) sections with a cryostat (CM3050 S, Leica, Wetzlar, Germany) and collected on SuperFrost Plus microscope slides (03–0060 Langenbrinck, Emmendingen, Germany). If not used immediately, tissue sections were stored at -80 °C until needed.

### Immunohistochemistry

For detection of patient aAb, sections were fixed on ice with ice-cold PFA (2% in PBS pH 7.4) for 30 s and then dipped into 50 mM NH_4_Cl for quenching before 30 min incubation with 0.1 mM glycine at RT. Next, sections were washed three times in PBS pH 7.4 in a staining dish, then blocked with 10% NGS in PBS for 1 h at RT. For co-staining of patient sera (diluted 1:100 in 5% NGS in PBS) with commercial mouse-anti-GlyRα1antibody mAb2b (1:500; 146111; Synaptic Systems) and rabbit-anti-synaptophysin (1:500; AB9272, Merck, Darmstadt, Germany), the tissue sections were incubated with the mix over night at 4 °C. Slices were washed three times in PBS for 10 min each, incubated with secondary antibodies (all from Jackson Immunoresearch, Cambridgeshire, UK) goat-anti-human-IgG-Cy3 (109-165-003), goat-anti-mouse-IgG-Alexa647 (115-605-003) and donkey-anti-rabbit-IgG-Alexa488 (711-545-152) diluted 1:500 in 5% NGS in PBS for 1 h at RT. Cell nuclei were stained with DAPI in PBS for 10 min before a final washing step in PBS. Slices were then dipped in H_2_O_dest_ before mounting with FluorSave Reagent (345789, Merck).

Staining of pan-GlyRα with a commercial antibody required a different protocol including antigen retrieval: After thawing at RT for 20 min and washing in PBS pH 8.0 for 5 min, the tissue was fixed with 2% PFA in PBS pH 8.0 for 10 min at 4 °C. For antigen retrieval, samples were washed twice for 5 min each (PBS pH 8.0, RT), incubated with 10 mM citrate buffer (10 mM tri-sodium citrate dehydrate and 0.05% Tween-20 in PBS pH 8.0) at 85 °C in a water bath for 30 min. After letting them air dry at RT for 20 min, slices were rehydrated for 5 min in PBS pH 8.0 and then blocked and permeabilized with 10% NGS and 0.3% Triton-X_100_ in PBS pH 8.0 before overnight incubation with the commercial primary antibodies mAb4a (anti-GlyR-pan-α, mouse monoclonal, dilution 1:250) and rabbit-anti-synaptophysin (1:500) in 5% NGS in PBS pH 8.0. As secondary antibodies, goat-anti-mouse-Alexa647 (1:500; 115-605-003,) and donkey-anti-rabbit-Alexa488 (1:500, 711-545-152) were used.

### Image analysis

Images of cultures cells were taken with a confocal Olympus Fluoview ix1000 microscope (Olympus, Hamburg, Germany) using an UPLSAPO 60x oil objective, images of tissue binding with a Zeiss Axio Imager 2 microscope (Zeiss, Jena, Germany) with a 20x air objective. All images were processed for analysis with the Fiji/ImageJ Software [35].

## Results

Twelve GlyR aAb positive patients were assigned into three groups according to their primary diagnosis (Table 1): SPS (*n* = 7 patients), PERM (*n* = 2) and epilepsy (*n* = 3).

**Fig. 1:**
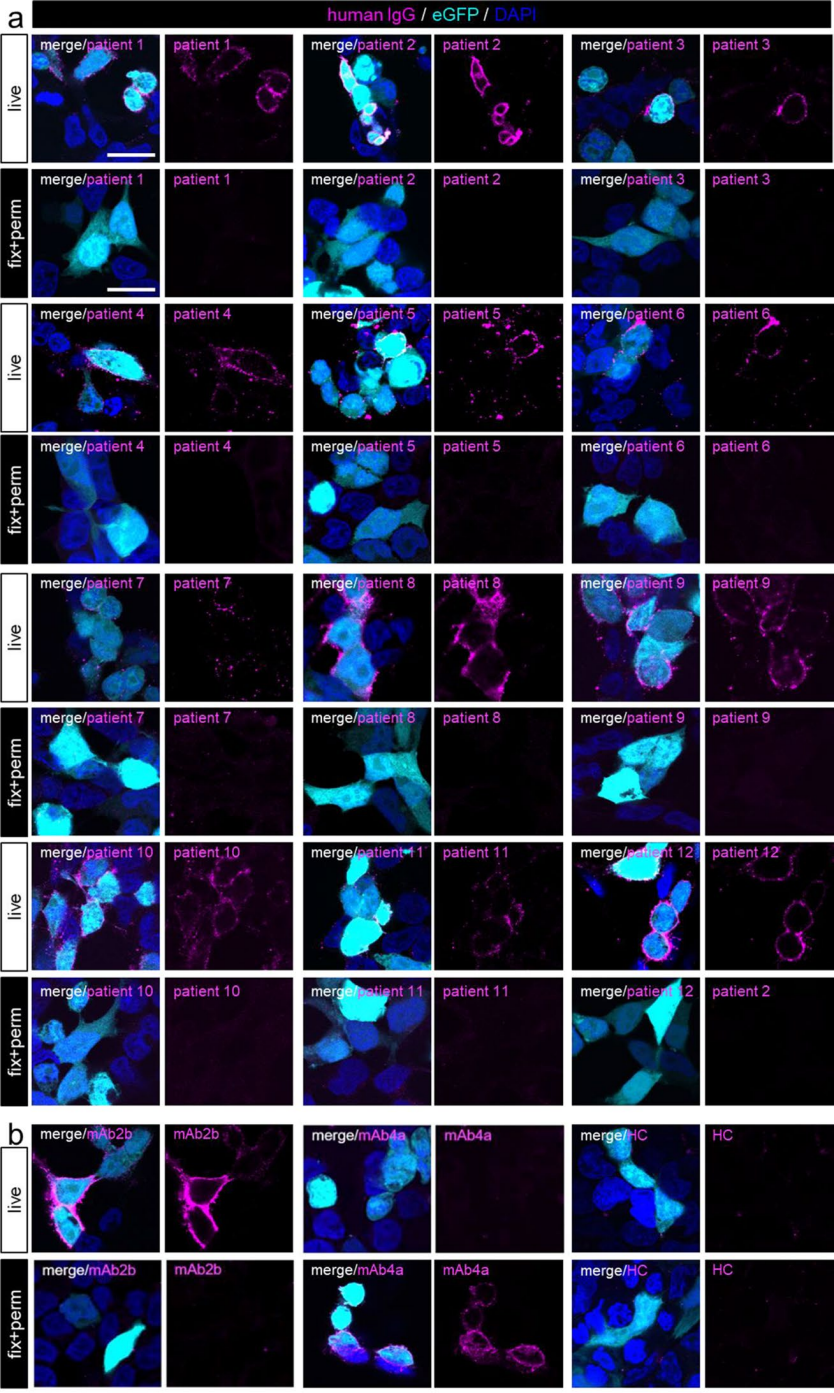
Patient GlyR aAb bind to living GlyRα1 transfected HEK-293 cells. **a**) Immunocytochemical stainings of all patient sera tested (patients 1 to 12, magenta) to GlyRα1 and eGFP (cyan) expressing HEK-293 cells. **b**) Control stainings with mAb2b staining GlyRα1 specifically under native conditions, while the pan-GlyRα antibody mAb4a binds fixed and permeabilized cells (magenta) only. Healthy control serum (HC) did not show staining. Cell nuclei are stained with DAPI (blue). Scale bar = 20 μm. Note, all patient sera stained GlyRα1 transfected HEK-293 cells only under native conditions

Before tissue-based analysis, serum samples were investigated for GlyR binding on transfected HEK-293 cells using live-cell staining. GlyRα1 has been demonstrated to be the GlyR subunit mostly targeted by aAbs [5, 32, 41]. Thus, the GlyRα1 subunit was co-transfected with eGFP. The latter served as a transfection control. For all samples tested, staining on living cells revealed specific aAb binding to GlyRα1 in eGFP-positive cells (Fig. 1a) arguing for binding to the extracellular domain of GlyRα1. As a positive control, the commercial GlyRα1-specific antibody mAb2b was used. Serum from a healthy individual (HC) served as a control to exclude unspecific binding (Fig. 1b). Binding to other GlyRα subunits, GlyRα2 and GlyRα3, was also determined by cell-based assays (Table 1). Some but not all patient sera bind GlyRα2 or GlyRα3 in addition to GlyRα1.

All GlyR aAb-containing sera were also tested for binding on fixed and permeabilized transfected HEK-293 cells to label possible intracellular epitopes. Here, a pan-GlyRα antibody (mAb4a) was used as positive control. In contrast to staining on living cells, no serum binding was observed when using the fixed HEK-293 cells (Fig. 1a). Therefore, GlyR aAb binding requires most likely the native receptor protein. The challenge in immunohistochemical stainings was to circumvent long fixation steps. We developed an appropriate staining procedure using unfixed fresh frozen tissue sections, a very short minor fixation step of 30 s with 2% PFA which then still allowed subsequent binding of patient aAbs.

### GlyR aAb binding to CNS regions rich in GlyRs - spinal cord and hindbrain

**Fig. 2:**
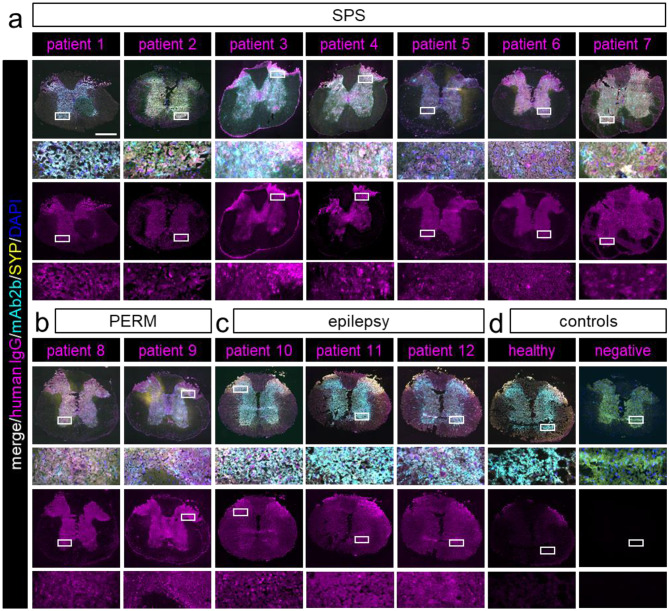
GlyR aAb binding to murine spinal cord tissue co-localizes with specific GlyRα1 labeling. **a**) Representative immunohistochemical labeling of murine spinal cord tissue sections with SPS, **b**) PERM, **c**) epilepsy patient serum samples, **d**) controls (magenta) co-stained with the commercial anti-GlyRα1 antibody mAb2b (cyan), synaptic marker synaptophysin (SYP; yellow) and DAPI (nucleus; blue). Each patient sample was tested in at least three independent experiments (*n* = 3). For each patient, the merged image as well as the patient channel alone (magenta) are displayed. Enlargements focus on dorsal horn for patients with specific aAb binding in this area and ventral horn for all other patients. Scale bar overview images = 500 μm, enlargements = 100 μm

GlyR aAb have been shown to bind to the extracellular domain (ECD) of the GlyR [32]. The ECD is highly homologous between human and mice (> 99.5% homology), making murine tissue a suitable tool to investigate patient aAb binding patterns. GlyRα1 is enriched in the grey matter of the adult spinal cord. Patient sera were thus first tested for binding patterns on murine spinal cord sections (Fig. 2).

Sera from all SPS (patients 1 to 7) and PERM (patients 8, 9) patients caused specific immunolabeling of the spinal cord grey matter (Fig. 2a and b). Co-labeling of patient sera with the commercial GlyRα1-specific antibody mab2b argues for binding of patient aAbs to GlyRs in these areas of tissue sections. In some cases, the extensive staining was accompanied by labeling of disperse single cell bodies. Such staining of higher intensity was present in dorsal laminae (patients 1, 3, 4, 9, and 10), the ventral horn (homogenous staining for all patients), and around the central canal (patients 3, 4, and 9) (enlargements of Fig. 2a-c). Serum samples from epilepsy patients also stained the spinal cord, although with lower intensity and without difference between grey and white matter. The signals of synaptophysin and GlyRα1 (mAb2b) did not co-localize with the human IgG from epilepsy patients in the white matter, indicating that staining in these areas was not caused by GlyR-specific aAb (Fig. 2c, patients 10–12). On all tested murine tissues, incubation with healthy control serum caused a low intensity background staining while the negative control lacked specific signals (Fig. 2d).

**Fig. 3:**
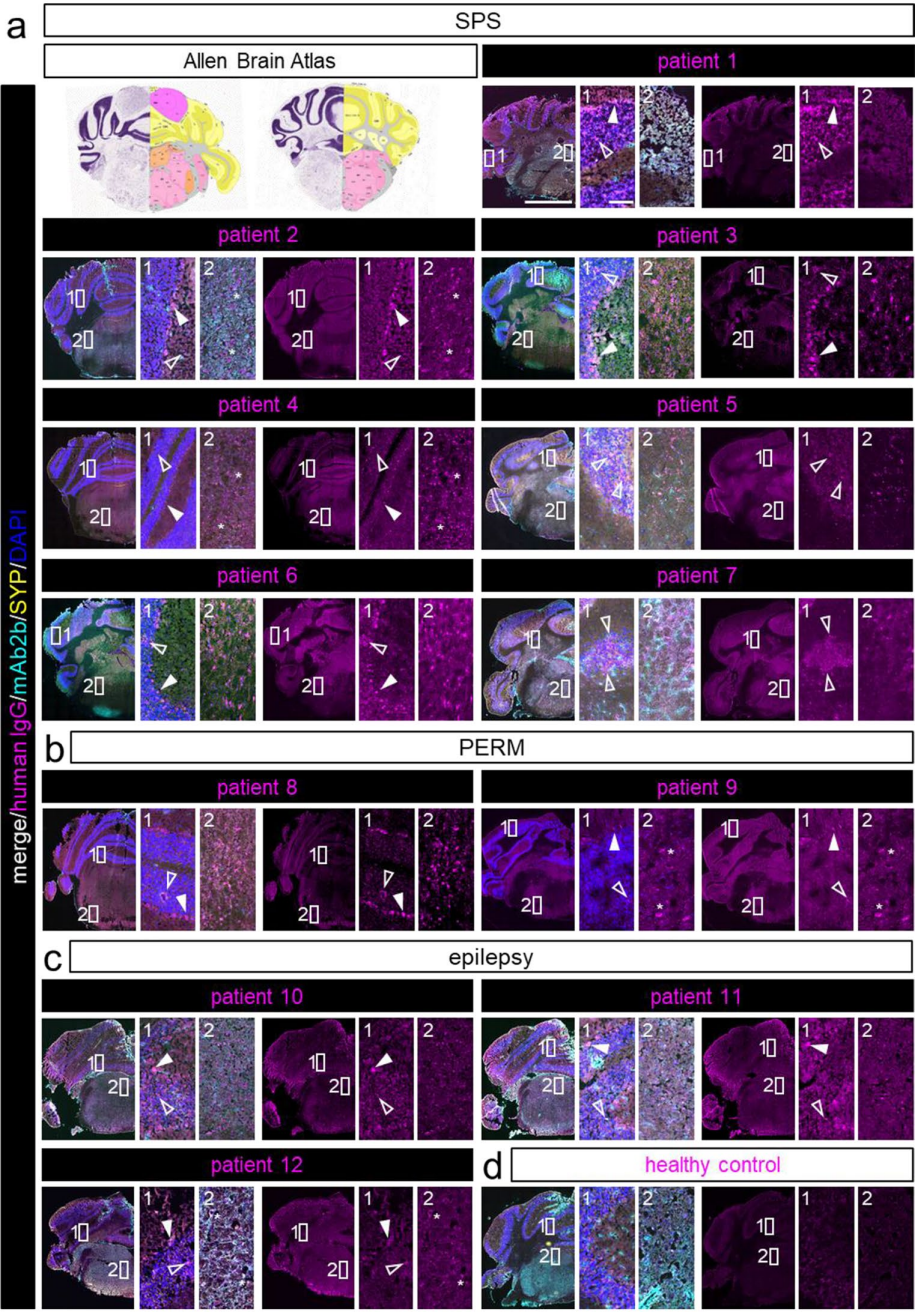
GlyR aAb show specific binding to GlyR-rich areas and cells layers of cerebellum and brainstem. aAb from patients’ serum samples bind to various areas of hindbrain tissue sections. aAb staining (magenta) of patients with **a**) SPS, **b**) PERM, and **c**) epilepsy is accompanied by labeling of GlyRα1 (cyan), synaptophysin (SYP; yellow) and cell nuclei (DAPI; blue), **d**) healthy control. For each patient, a merge of all stainings and the according patient staining are displayed. Enlargements show most intensive patient autoantibody labeling within cerebellum (box 1) and brainstem (box 2). Empty arrow heads point toward granule cell staining, filled arrow heads indicate Purkinje cell labeling. * in enlargement images in box 2 refer to specific patient aAb staining in nuclei of visual or auditory pathways for individual patients. Scale bar in overview images = 2000 μm; scale bar in enlargement refers to 100 μm. Nissl (left) and anatomical annotations (right) from the Allen Mouse Brain Atlas are included for brain area reference

Besides the spinal cord, GlyRs are mainly expressed in the brainstem [3], which makes this area of network integration by various nuclei a common target for GlyR directed aAb. About half of the tested SPS patients’ samples caused immunolabeling of brainstem nuclei. Similar staining was detected for both PERM patients and two of the three epilepsy patients. Immunolabeling of various brainstem nuclei was detected for all patients (Fig. 3a-c, box 2). The medulla as part of the brainstem controls autonomic muscle activity for vital processes such as respiration and heartbeat [22]. Here, about 70% of the tested SPS, one of the PERM, and all three epilepsy patients showed specific antibody binding (Fig. 3a-c, box 2). In the cerebellum, serum from all tested SPS, PERM and epilepsy patients specifically labeled the granule cell layer (empty arrow heads in enlargements in Fig. 3a-c, box 1). Additionally, samples from all epilepsy and PERM patients as well as most SPS patients stained the GlyR expressing Purkinje cells (filled arrow heads in enlargement in Fig. 3a-c, box 1), which contribute to movement coordination, cognition and emotion.

### GlyR aAb binding patterns in higher brain areas

**Fig. 4:**
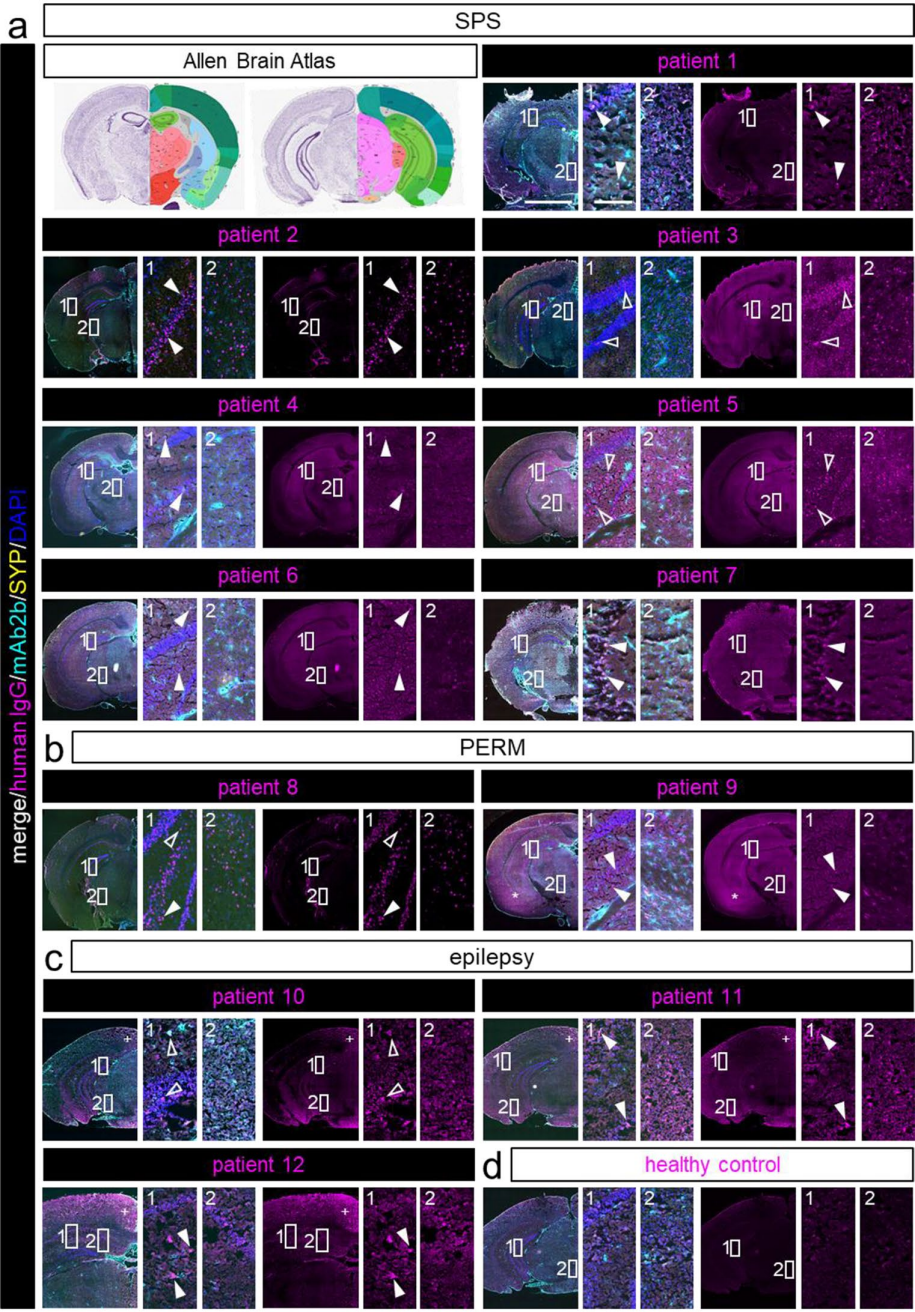
GlyR aAb bind to murine midbrain tissue sections. aAb from patient serum samples bind to areas of midbrain and cerebral mouse tissue sections in immunostainings. Human IgG is shown in magenta, mAb2b labels GlyRα1 (cyan), synaptophysin serves as a synaptic marker (SYP; yellow) and nuclei are stained with DAPI (blue). For each patient with **a**) SPS, **b**) PERM, **c**) epilepsy the merge of mAb2b, SYP, and DAPI stainings with the corresponding human IgG are displayed, **d**) healthy control. Enlargements show layers of the hippocampal formation (box 1) and thalamus or hypothalamus (box 2) where specific aAb staining was detected. Empty arrow heads point toward dentate gyrus granule cell staining, filled arrow heads indicate CA1/2/3 pyramidal cell labeling. * and + in overview images refer to specific patient aAb staining in the amygdala for patient 9 or the retrosplenial area for patient 10–12, respectively. Scale bar in overview images = 2000 μm, scale bar in enlargement = 200 μm. Nissl (left) and anatomical annotations (right) from the Allen Mouse Brain Atlas are included for brain area reference. Note, there are differences in staining intensity and distribution between groups and patients within groups

The hippocampal formation was of special interest for GlyR aAb binding of epilepsy patients. The hippocampus does however not exhibit extensive GlyR expression [24]. The GlyRα3L splice variant was shown preferentially associated with glutamatergic nerve endings in strata granulare and pyramidale and as functionally relevant in epilepsy. In patients with temporal lobe epilepsy, the short GlyRα3K splice variant which is more diffusely distributed is however upregulated in expense of GlyRα3L [6, 15, 16]. As displayed in Fig. 4, all tested patient samples caused staining of some cell layers of the hippocampal formation. For SPS patients, the staining pattern of granule cells and CA1 or CA3 pyramidal cells varied, but each cell layer was stained in more than half of the tested patients (five out of seven; filled arrow heads in enlargement images of Fig. 4a, box 1). In contrast, both PERM patients’ serum bound to all three cell layers (arrow heads in enlargement images of Fig. 4b, box 1). Serum from epilepsy patients mostly bound to the granule cells of the dentate gyrus (empty arrow heads in enlargements of Fig. 4c, box 1) and CA1 pyramidal cells (filled arrow heads in enlargement images of Fig. 4c, box 1). In addition, all three epilepsy patients’ serum also stained the subiculum.

Further stained areas included midbrain nuclei and certain cortical areas as well as regions in the thalamus and hypothalamus (Fig. 4a-c, box 2). However, immunolabeling in these areas was scarce and rather individual between patients than uniform among the different groups. Additionally, patient 9 showed intense labeling in the region of the amygdala.

### GlyR aAb mainly target GlyRα1 positive brain regions

**Fig. 5:**
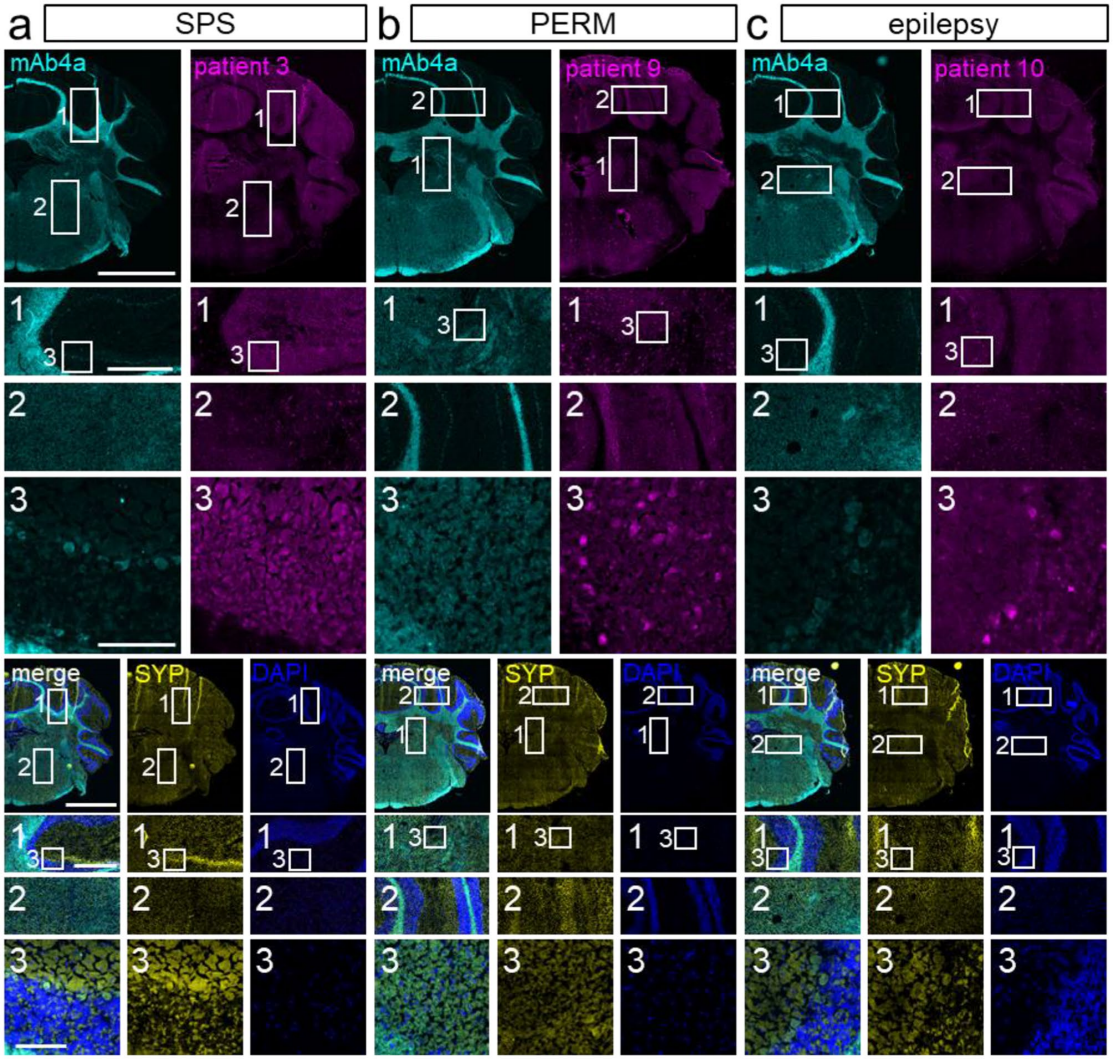
Immunohistochemical analysis of hindbrain tissue with GlyR aAb and pan-α GlyR labeling. Fluorescent labeling displays aAb binding in GlyRα rich brain areas. For each tissue section stained with patient serum (magenta) and DAPI (blue) with **a**) SPS, **b**) PERM, **c**) epilepsy the next neighboring section was labeled for GlyR with a pan-α antibody (mAb4a; cyan), synaptophysin (SYP; yellow) and nuclei (DAPI; blue). In addition to the single channels, mAb4a and synaptophysin are displayed in a merge with DAPI. Distinct tissue sections’ enlargements (box 1, cerebellum; box 2 brainstem; box 3 enlargement of box 1) were positioned as similar as possible. For each patient group, images of one representative patient are shown. Scale bars: overview images = 2000 μm, first enlargements = 500 μm, second enlargement = 100 μm

**Fig. 6:**
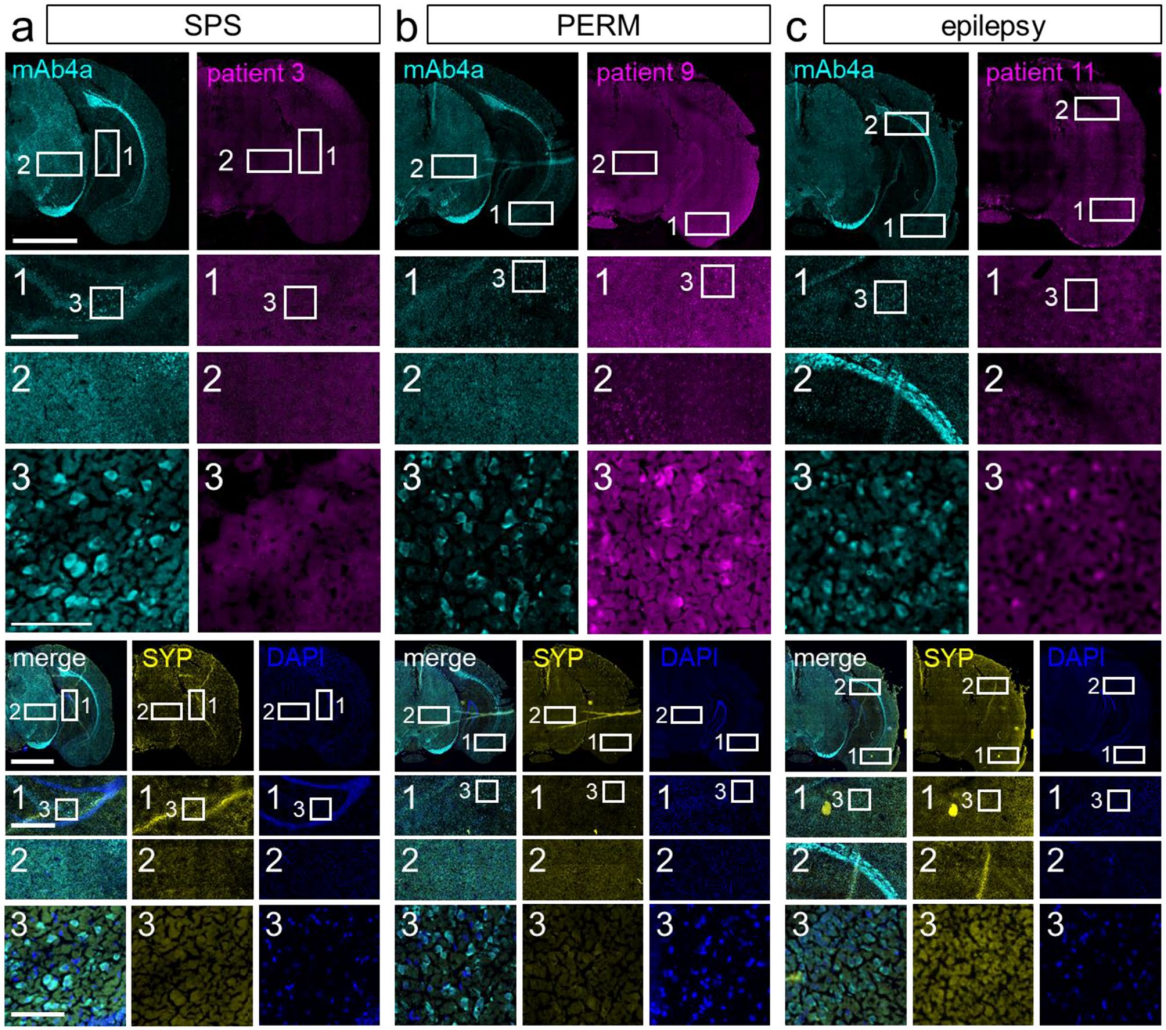
Midbrain tissue sections display labeling of GlyR aAb and all GlyRα in parallel stainings. Patient serum and DAPI labeling were performed in the same section with **a**) SPS, **b**) PERM, and **c**) epilepsy, while GlyRα (mAb4a; cyan), synaptophysin (SYP; yellow) were stained on the next neighboring tissue section. The merge shows mAb4a, synaptophysin and DAPI labeling. Areas (box 1 hippocampus, box 2 additional specific aAb staining, box 3 represents an enlargement of box 1) displayed in the enlargements of the distinct tissue sections were chosen as similar as possible. For each group, images of one representative patient are shown. Scale bars: overview images = 2000 μm, first enlargements = 500 μm, second enlargement = 100 μm

GlyR aAb binding in the spinal cord and hindbrain mostly co-localized with GlyRα1 staining (Fig. 2), but GlyR staining in higher brain areas was scarce due to the minor presence of GlyRα1 in those regions. To exclude misinterpretation of binding of GlyR aAbs to higher brain areas with a more abundant expression of other GlyRα2 or GlyRβ, brain tissue sections were stained with the pan-α antibody mAb4a that labels other GlyRα subunits e.g. α2, α3 and to some extent GlyRβ. GlyRα2 and GlyRβ i.e. are more prominent in higher brain areas than GlyRα1 [2, 24, 28, 40]. The pan-α antibody mAb4a requires tissue fixation and antigen retrieval. GlyR aAbs are however sensitive to fixation as indicated by the lack of specific staining on GlyRα1 transfected HEK-293 cells fixed and permeabilized (Fig. 1). To circumvent this problem, mAb4a, synaptophysin and patient serum staining was performed in serial sections. This procedure did not allow co-staining of exactly the same cells but facilitated parallel GlyR and aAb labeling of cells in the same brain areas and cell layers. One representative example per patient group is displayed for hindbrain (Fig. 5a-c) and for cerebral cortex, hippocampus, thalamus and hypothalamus (Fig. 6a-c). Interestingly, no additional brain areas were intensively targeted by patient aAb compared to previous co-labeling with GlyRα1 arguing that we did not miss specific labeling of GlyR aAbs in other higher brain areas. However, whether the aAb labeling is due to exclusively GlyRα1 binding or a mixture of different GlyR configurations cannot be totally resolved.

## Discussion

**Fig. 7:**
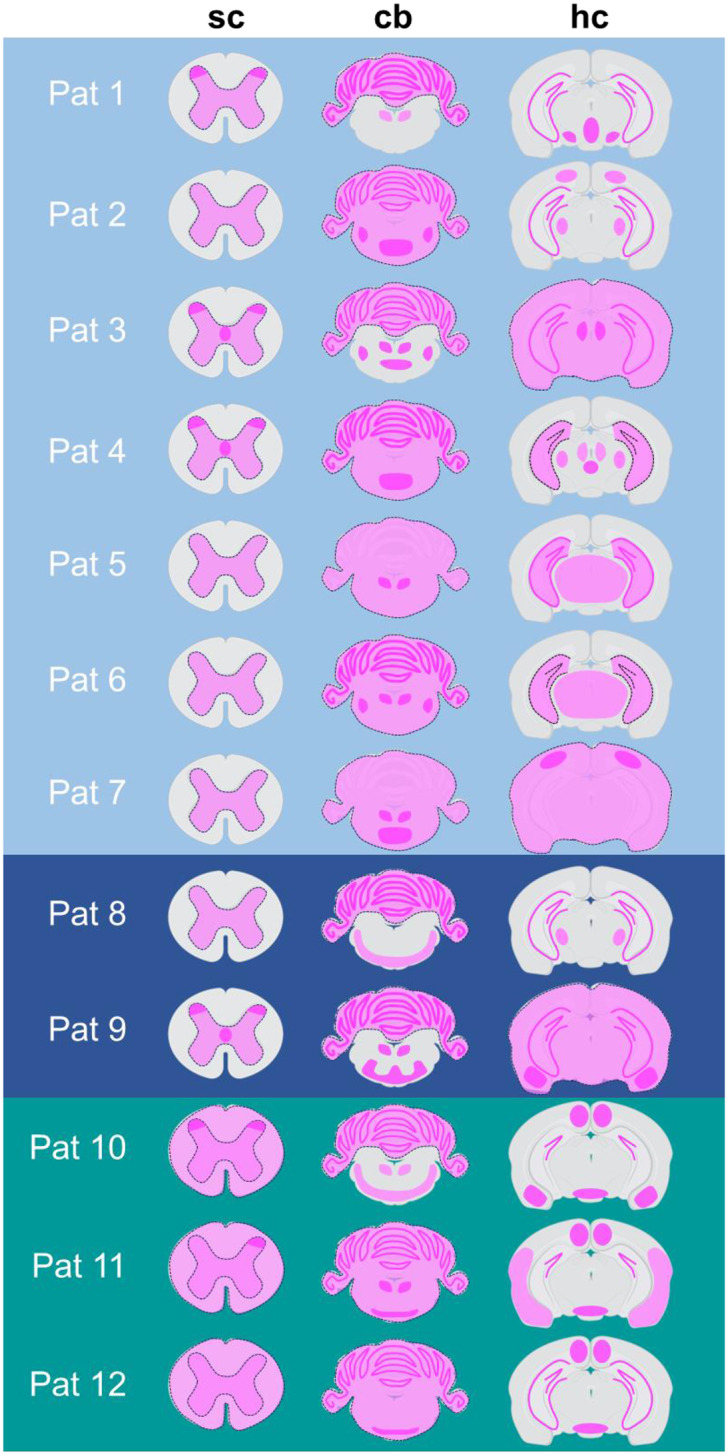
Overview of patient binding patterns. Scheme of individual patient binding patterns to different CNS regions (sc = spinal cord, cb = cerebellum, hc = hippocampus region). Binding strength is indicated via light and dark magenta labelling

GlyR aAbs are associated with neurological disorders, including SPS and PERM, epilepsy and encephalitis. GlyR aAbs bind mainly to the GlyRα1 subunit but in some cases to other GlyRα or even β subunits [5, 41]. Why aAbs to the same target induce such different diseases, is as yet unknown. At the molecular level, common pathological mechanisms such as receptor crosslinking and subsequent internalization or direct blocking of GlyR function upon binding have been demonstrated [9, 32]. Here we tried to identify binding patterns of patient serum on murine spinal cord and brain, which potentially might explain the different symptoms (Fig. 7; Table 2). We found several patterns distinguishing the different syndromes: (1) Specific GlyR binding in the grey matter of the spinal cord was only present in patients with SPS and PERM, not in epilepsy. (2) Only for PERM patients all sera bound to all three layers of the hippocampus, (3) Only serum from the epilepsy patients distinctly bound to the subiculum.

**Table 2:**
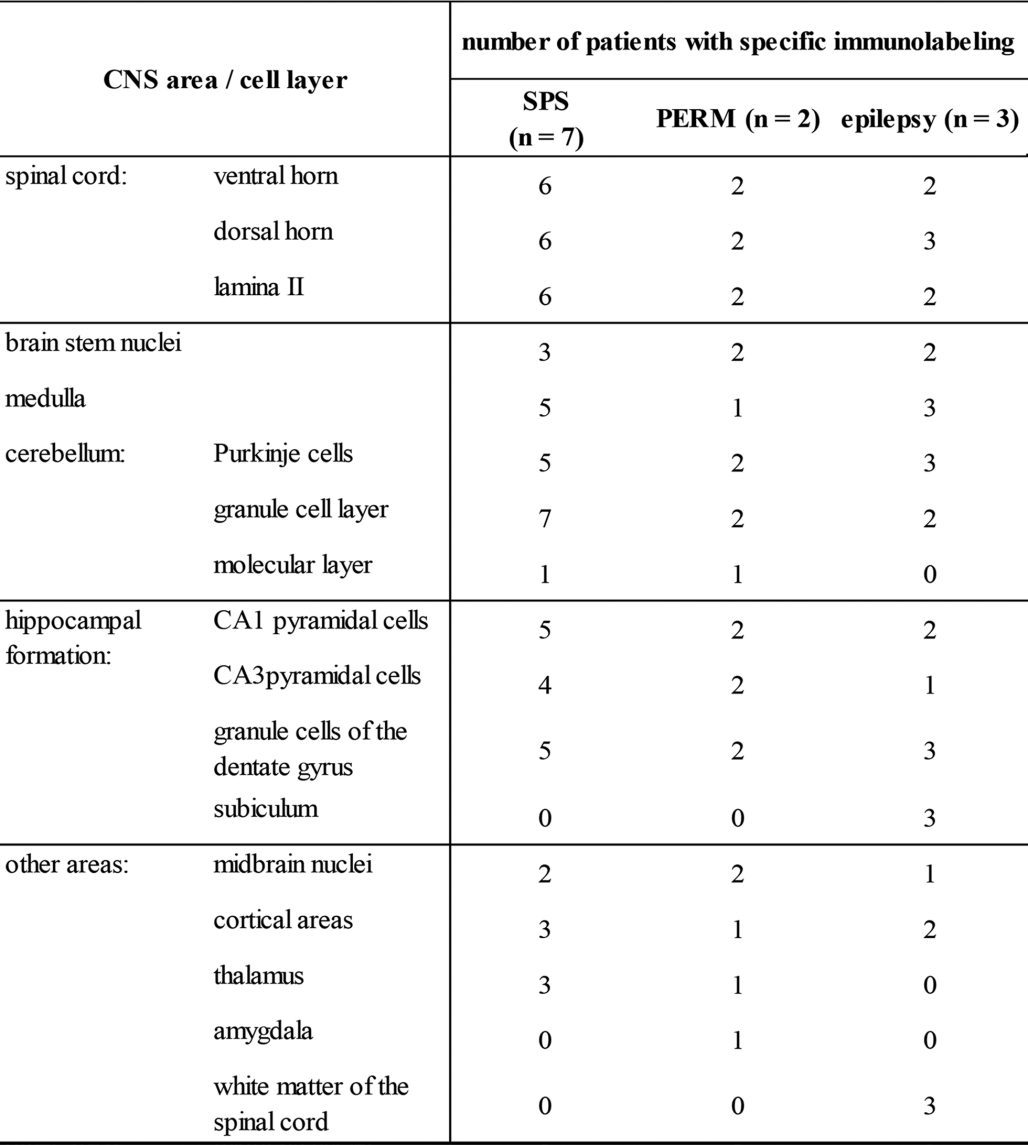
Patient autoantibody binding patterns

In 2014, Carvajal-Gonzalez et al. described GlyR aAb binding in the spinal cord and brainstem but also hippocampus and cerebellum for GlyR aAb positive patients and suggested a relation between the involvement of these regions and the patients’ disease characteristics [5]. However, the relation of binding patterns and individual symptoms has not been studied so far, nor has the association between binding patterns and the different diseases (SPS, PERM, epilepsy) related to GlyR aAbs.

For SPS and PERM patients, the most prominent binding was detected in the grey matter of the spinal cord, where the large motoneurons and glycinergic inhibitory interneurons accumulate. This supports the notion of aAb mediated interference with inhibitory signaling causing the strong motor phenotype of spasms and stiffness in the limbs observed in these patients. Motor symptoms in SPS and PERM patients might however not be limited to pathomechanisms in the spinal cord. Various brain areas involved in motor behavior harbor GlyRs as well [23, 46]. Hence, GlyR directed aAb binding in the brain possibly contributes to the effects caused by these aAbs.

Similar to the motor symptom associated labeling of the spinal grey matter, aAbs from SPS and PERM patients additionally stained the motor pathway related medulla as well as cerebellar Purkinje and granule cells. These cell groups contribute to information processing and movement coordination [1, 24], which aligns these autoantibody deposits with the patients’ motor impairments and gait disorders. Sudden falls might result from insufficient coordination upon excessive startle responses. In addition, single patients of each group displayed aAb deposition in areas involved in visual and auditory pathways (* in enlargement images of Fig. 3a-c, box 2), to which GlyR activity strongly contributes [38]. However, visual symptoms have only been reported in one SPS patient (patient 2).

Many GlyR aAb positive patients have symptoms beyond the typical motor impairment. Especially in PERM patients, symptoms often include anxiety, brainstem dysfunction, myoclonus or seizures [5, 11, 12].

In higher brain regions, autoantibodies from many of the SPS patients bound to the dentate gyrus granule cells as well as the CA1 and CA3 pyramidal cells. The hippocampus is involved in contextual memory retrieval as well as context-dependent fear extinction [20]. Thus, GlyR aAb binding in this area might promote enhanced anxiety or phobic behavior. Even though the staining intensity does not appear in proportion to symptom severity, for one of the PERM patients (patient 9) hippocampal staining was accompanied by significant autoantibody labeling of regions in the amygdala (* in overview images of patient 9, Fig. 4b). Patient 9 was diagnosed with noticeably strong psychiatric features including depression and anxiety disorder. This might add to the notion of aAb evoked anxiety behavior due to hippocampal aAb binding [44]. Dysfunction of hippocampal glycine signaling is associated with neuropsychiatric disorders [43], but the specific role of GlyRs in the hippocampus is still not fully elucidated.

Interestingly, clinical features of PERM patients may also include seizures. As the hippocampal formation is known for its crucial role in epilepsy [6], autoantibody binding in this area underpins the assumption that PERM patients’ seizures may be GlyR pathology-related and also points out parallels between the different GlyR aAb associated diseases [48]. Thus, the group of epilepsy patients was expected to reveal greatest aAb signal in the hippocampus. Autoimmune-mediation is suspected to underlie epilepsy of unknown cause [14, 19], which was the primary diagnosis for patients 10 and 11. But, even if present, the GlyR autoantibody signal for the epilepsy patients in this area was surprisingly low. Our study demonstrated that only serum from epilepsy patients distinctly bound to the subiculum, a pivotal output component of the hippocampus. Notably, accumulating evidence from both clinical and experimental studies suggests that the subiculum plays a vital role in seizure initiation and propagation in epilepsy [4].

Interestingly, aAb binding in epilepsy patients was also detected in the spinal cord (gray and white matter), where it was rather unexpected as the epilepsy patients do not present with SPS-like motor symptoms. Since the white matter binding was not co-labeled with specific GlyR antibodies, we assume that the patient serum harbor additional unidentified aAbs which allowed white matter labeling. For example, myelin oligodendrocyte glycoprotein directed aAbs were previously mentioned in the context of seizures [18]. Conversely, the staining of myelinated areas was not detectable in brain sections. Instead, aAbs from all tested epilepsy patients caused overall extensive staining of the brain sections that co-localized with mAb4a labeling of expressed GlyRs and left out the myelinated fiber tracts. This supports GlyR-specific aAb binding to the brain sections, with a possible causative connection to seizure generation. Higher brain areas express rather no GlyRα1, but GlyRα2. The determined positive binding of aAbs from epilepsy patients to GlyRα2 and/or α3 in cell-based assays substantiates the finding of binding to higher brain areas which express those receptor subunits. Furthermore, aAb labeling in epilepsy patients was present in the interpendicular nucleus, the retrosplenial area (+ in overview images of patient 10–12, Fig. 4C) and the midbrain substantia nigra. These areas contribute to autonomic and cognitive functions, respectively [37]. The relation between autonomic function and seizures however seems to go both ways, as cognitive functions are more likely impaired by recurrent epileptic seizures [7, 30]. Thus, aAb mediated signal imbalances in related areas might contribute to seizures as a characteristic disease pathology in PERM and epilepsy patients.

Overall, GlyR directed aAbs from patients with different GlyR related syndromes all bound GlyR rich areas of the CNS.

### Limitations

Amino acid differences between human and rodent proteins may have an impact on aAb binding [26, 33]. However, the amino acid sequences of human and murine GlyR subunits share a high degree of homology with only some exchanges in the extracellular domain representing the target for aAb binding [32]. Furthermore, the staining procedure of other GlyR subunits except α1 and the comparison to the aAb staining did not allow testing on the same tissue section of the brain. And, due to the rareness of the disease, sample sizes of patients in each disease group limit group-specific conclusions in the context of diagnostics and treatment.

## Conclusions

In summary, the obtained binding pattern of GlyR aAb containing patient sera to distinct CNS areas are related to the diverse clinical representations of the patients. Further research should look into the functional changes induced by aAbs in the different CNS areas.

## Data Availability

No datasets were generated or analysed during the current study.

## Abbreviations

ECD: Extracellular domain
GlyR: Glycine receptor
SPS: Stiff person syndrome
PERM: Progressive encephalomyelitis with rigidity and myoclonus
Pat: Patient
aAb: Autoantibody
